# Browning of Mammary Fat Suppresses Pubertal Mammary Gland Development of Mice via Elevation of Serum Phosphatidylcholine and Inhibition of PI3K/Akt Pathway

**DOI:** 10.3390/ijms242216171

**Published:** 2023-11-10

**Authors:** Limin Lang, Jisong Zheng, Shuyi Liang, Fenglin Zhang, Yiming Fu, Kaixin Deng, Fan Li, Xiaohua Yang, Junfeng Wang, Yuexiang Luo, Shilei Zhang, Xiaotong Zhu, Lina Wang, Ping Gao, Canjun Zhu, Gang Shu, Qianyun Xi, Yongliang Zhang, Qingyan Jiang, Songbo Wang

**Affiliations:** Guangdong Provincial Key Laboratory of Animal Nutrition Control, National Engineering Research Center for Breeding Swine Industry, College of Animal Science, South China Agricultural University, Guangzhou 510642, China; langlanglimin@163.com (L.L.); zhengjs1213@163.com (J.Z.); 15099964676@163.com (S.L.); zhangfenglin@scau.edu.cn (F.Z.); fuyiming396@163.com (Y.F.); m18039175360@126.com (K.D.); kyanzedd@163.com (F.L.); yangxiaohua137@163.com (X.Y.); wjf1998777@163.com (J.W.); 17666548138@163.com (Y.L.); zsl611@stu.scau.edu.cn (S.Z.); xtzhu@scau.edu.cn (X.Z.); wanglina@scau.edu.cn (L.W.); gaoping@scau.edu.cn (P.G.); canjunzhu@scau.edu.cn (C.Z.); shugang@scau.edu.cn (G.S.); xqy0228@163.com (Q.X.); zhangyl@scau.edu.cn (Y.Z.); qyjiang@scau.edu.cn (Q.J.)

**Keywords:** browning of mammary fat, mammary gland development, phosphatidylcholine, proliferation, PI3K/Akt pathway

## Abstract

Mammary fat plays a profound role in the postnatal development of mammary glands. However, the specific types (white, brown, or beige) of adipocytes in mammary fat and their potential regulatory effects on modulating mammary gland development remain poorly understood. This study aimed to investigate the role of the browning of mammary fat on pubertal mammary gland development and explore the underlying mechanisms. Thus, the mammary gland development and the serum lipid profile were evaluated in mice treated with CL316243, a β3-adrenoceptor agonist, to induce mammary fat browning. In addition, the proliferation of HC11 cells co-cultured with brown adipocytes or treated with the altered serum lipid metabolite was determined. Our results showed that the browning of mammary fat by injection of CL316243 suppressed the pubertal development of mice mammary glands, accompanied by the significant elevation of serum dioleoylphosphocholine (DOPC). In addition, the proliferation of HC11 was repressed when co-cultured with brown adipocytes or treated with DOPC. Furthermore, DOPC suppressed the activation of the PI3K/Akt pathway, while the DOPC-inhibited HC11 proliferation was reversed by SC79, an Akt activator, suggesting the involvement of the PI3K/Akt pathway in the DOPC-inhibited proliferation of HC11. Together, the browning of mammary fat suppressed the development of the pubertal mammary gland, which was associated with the elevated serum DOPC and the inhibition of the PI3K/Akt pathway.

## 1. Introduction

Good development of the mammary gland in mammals is the key premise for improving lactation performance and providing nutrition for offspring. The postnatal development of mammary glands mainly includes several stages such as puberty, pregnancy, and lactation [1,2]. Among these, the pubertal development of mammary glands is particularly critical for the later function during pregnancy and lactation [3,4,5].

Mammary gland development is influenced by various factors, such as hormones, growth factors, nutrition, and so on [6]. It has been demonstrated that dietary supplementation of fatty acids plays an important role in regulating mammary ductal growth and the number of terminal end buds (TEBs) [4,7]. High-energy diets and feeding restrictions both elicited detrimental effects on mammary gland development in sows [8,9]. In addition, the morphogenesis and functional differentiation of the mammary epithelium also depend on some internal factors like the local microenvironment of the tissue and the cell–cell interaction [10,11]. 

The mammary gland is composed of parenchymal tissue and extraparenchymal tissue, with the parenchymal tissue consisting of ducts and alveoli [12]. The extraparenchymal tissue is mainly composed of connective tissue and adipose tissue [13]. As the major component and contributor to the volume of the mammary gland, adipose tissues play a profound role in the postnatal development of the mammary gland [14,15]. When entering the pubertal stage, an adipocytes-filled fat pad consists of a large proportion of the mammary gland; thus, ducts expand into the fat pad under the stimulation of hormones [16]. Karen et al. found that there was no potential for the endogenous epithelium to extend mammary ductal trees when the mammary fat pad was removed at 3 weeks [17]. Similarly, gilts with less total mammary fat showed a detrimental effect on mammary gland development [5]. Interestingly, the increased adiposity was also associated with stunted ductal growth and mammary epithelial cell proliferation in pubertal mice [18]. These findings confirmed the possibility that adipose tissues in mammary fat exert vital effects on mammary gland development. Nevertheless, the specific mechanisms whereby adipose tissues regulate mammary glands are still poorly understood.

Previous studies have focused on the role of adipose tissue in regulating mammary gland development. However, little is known about the effects of specific types of adipocytes on modulating mammary gland development. Nowadays, adipocytes can be divided into three types: white, brown, and beige adipocytes. These three kinds of adipocytes differ in their anatomic structure and thermogenic capacity [14,19]. White adipocytes contain large unilocular lipid droplets and release fatty acids during the fasted state [14,20]. In contrast, brown adipocytes have much smaller multilocular lipid droplets and abundant mitochondria, burning lipids to thermogenesis by activating the uncoupling protein 1 (UCP1) [20]. Beige adipocytes show the phenotypes of both white adipocytes and brown adipocytes under different conditions [21,22]. Additionally, beige adipocytes are highly inducible from white adipocytes when exposed to chronic cold exposure or other inducing agents [20,23]. Previous perspectives assumed that mammary gland fat only consists of white adipocytes, while later data showed that both white and brown adipocytes are present in the postnatal mammary gland [21]. Furthermore, studies showed that unilocular white adipocytes could be consistently detected in mammary glands before and during puberty, while multilocular brown adipocytes were abundant in 2- and 3-week-old mice and restricted to the specific area between the nipple and the lymph node of mammary glands in prepuberty [14]. These findings suggested that the different types of adipose tissues or adipocytes might elicit different effects on pubertal mammary gland development. However, the role of the browning of mammary fat on mammary gland development during puberty remains largely unknown.

Thus, this study was designed to investigate whether the browning of mammary fat affected the development of mammary glands and explore the underlying mechanisms. Our data showed that the browning of mammary fat suppressed the development of mammary glands during puberty in mice, accompanied by the elevation of serum dioleoylphosphocholine (DOPC) level. In addition, the proliferation of HC11 was repressed when co-cultured with brown adipocytes or treated with DOPC and the PI3K/Akt pathway was involved in the DOPC-inhibited proliferation of HC11. 

## 2. Results 

### 2.1. Brown Adipocytes in Mammary Fat Decreased with the Pubertal Development of Mice Mammary Glands

**Figure 1:**
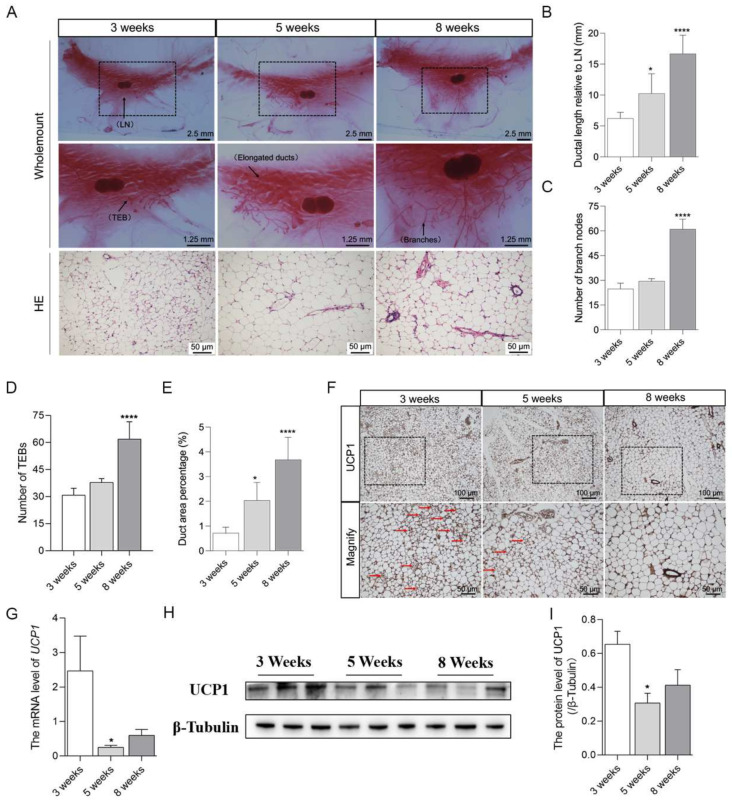
Brown adipocytes in mammary fat decreased with the pubertal development of mice mammary glands. (**A**) representative images of Wholemount staining (*n* = 6) and HE (*n* = 4) staining showed the changes in the morphology of mammary glands and characteristics of adipocytes in mammary fat from 3 weeks to 8 weeks. Black arrows indicate the lymph node (LN), terminal end bud (TEB), elongated ducts, and ductal branches respectively. The middle-row images of the figure represent the magnification of the dotted boxes in the up-row images. The analysis for the length of ducts (**B**), the number of branch nodes (**C**), the number of TEBs (**D**), and the duct area percentage (**E**) of mammary glands. (**F**) immunohistochemical staining for the expression of UCP1 along with the brown-like adipocytes as indicated by red arrows (*n* = 6). The down-row images of the figure represent the magnification of the dotted boxes in the up-row images. The mRNA (*n* = 6) (**G**) and protein (*n* = 6) (**H**) levels of UCP1 in the mammary gland. (**I**) the graph indicates densitometric analysis with expression ratios of UCP1/β-Tubulin. Values are presented as means ± SEM, * *p* < 0.05; **** *p* < 0.0001.

Firstly, the characteristics of adipocytes in mammary fat were investigated with the pubertal development of mice mammary glands from the age of 3 weeks to 8 weeks. As expected, the results of wholemount and HE staining demonstrated that the mammary gland has obvious development from 3 weeks to 8 weeks, with a significant increase in the length of ducts extended (Figure 1A,B), the number of ductal branches (Figure 1A,C), the number of terminal end buds (TEBs) (Figure 1A,D), and the ductal area percentage (Figure 1A,E). Interestingly, with the pubertal mammary gland development from 3 weeks to 8 weeks, the expression of UCP1 in mammary fat was markedly decreased as revealed by immunohistochemistry staining (Figure 1F). In addition, the brown-like adipocytes were in decline and the diameter of the adipocytes was elevated (Figure 1F). Consistently, the mRNA (Figure 1G) and protein (Figure 1H,I) levels of UCP1 were remarkably reduced from 3 weeks to 8 weeks. These findings demonstrated that the brown adipocytes in mice mammary fat decreased with the pubertal development of mammary glands and suggested the potential regulatory effects of brown or brown-like adipocytes in mammary fat on the pubertal mammary gland development. 

### 2.2. Pharmacological Induction of the Browning of Mammary Fat by Injection of CL316243

**Figure 2:**
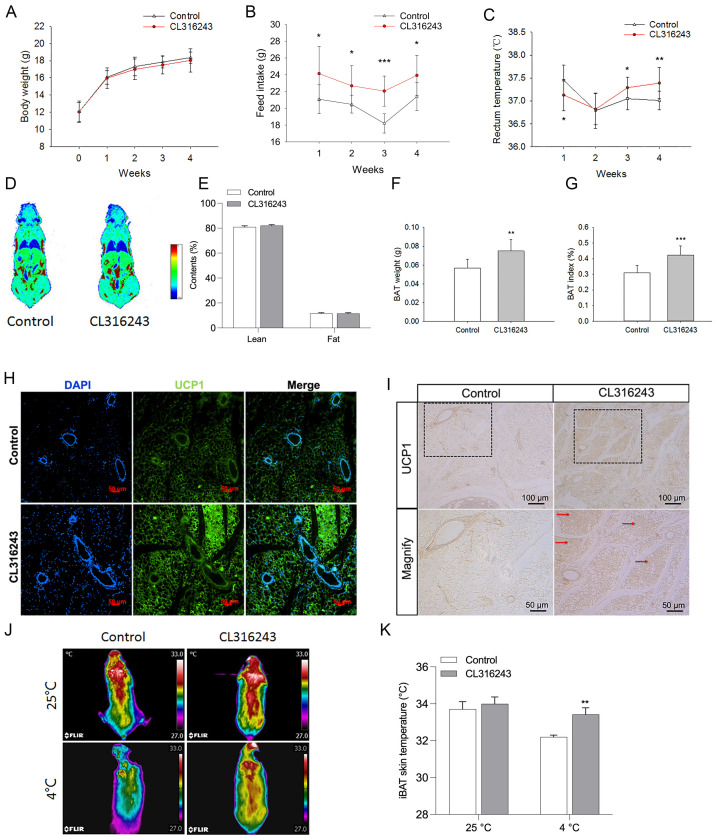
Pharmacological induction of the browning of mammary fat by injection of CL316243. Effects of injection of CL316243 on the body weight (**A**), food intake (**B**), rectum temperature (**C**), fat distribution (**D**), and lean and fat content (**E**) of mice (*n* = 10). The BAT weight (**F**) and BAT index (**G**) of mice treated with CL316243 (*n* = 10). Immunofluorescence staining (**H**) (UCP1: green, DAPI: blue) and immunohistochemical staining (**I**) showed the location and expression of UCP1 in mammary fat after the injection of CL316243 (*n* = 4). The down-row images of the figure represent the magnification of the dotted boxes in the up-row images. Brown-like adipocytes are indicated by red arrows. Representative images (**J**) and quantification (**K**) of interscapular BAT (iBAT) thermogenesis under an environmental temperature of 25 °C or a cold exposure temperature of 4 °C (*n* = 10). Values are presented as means ± SEM, * *p* < 0.05; ** *p* < 0.01; *** *p* < 0.001.

To elucidate the potential effects of brown or brown-like adipocytes in mammary fat on pubertal mammary gland development, the browning of mammary fat was pharmacologically induced by intraperitoneal injection of CL316243 into mice. We found that CL316243 treatment significantly increased the food intake of mice (Figure 2B), whereas had no effect on the body weight of mice (Figure 2A). In addition, the rectum temperature was elevated from 3 to 4 weeks by intraperitoneal injection of CL316243 (Figure 2C). These results implied the potentially elevated energy metabolism induced by CL316243. Although having no influence on body lean and fat contents (Figure 2D,E), the injection of CL316243 remarkably promoted UCP1 expression in the mammary fat (Figure 2H,I), indicating the browning of mammary fat. Furthermore, the brown adipose tissue (BAT) weight (Figure 2F) and BAT index (Figure 2G) were significantly increased by the CL316243 treatment. Moreover, the effect of CL316243 on BAT thermogenesis was analyzed by infrared thermography (Figure 2J). The results showed that the temperature of the interscapular BAT (iBAT) was significantly elevated by CL316243 treatment at 4 °C, with no difference at 25 °C (Figure 2K). Collectively, these findings indicated that CL316243 treatment induced the browning of mammary fat and the activation/thermogenesis of BAT.

### 2.3. Browning of Mammary Fat Suppressed the Pubertal Development of Mice Mammary Glands

**Figure 3:**
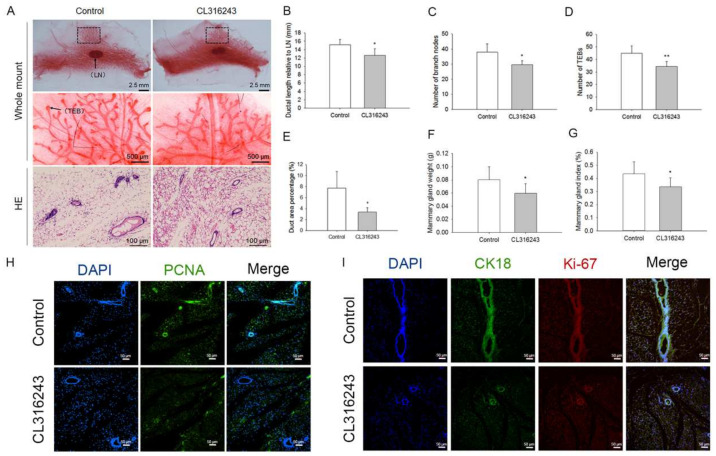
Browning of mammary fat suppressed the pubertal development of mice mammary gland. (**A**) representative images of Wholemount (*n* = 10) and HE (*n* = 5) staining of 8-week-old mice mammary gland after the injection of CL316243. Black arrows indicate the lymph node (LN) and terminal end bud (TEB) respectively. The middle-row images of the figure represent the magnification of the dotted boxes in the up-row images. Effects of the browning of mammary fat on ductal length (**B**), the number of branch nodes (**C**) and TEBs (**D**), duct area percentage (**E**), and the weight (**F**) and index (**G**) of mammary gland. Immunofluorescence staining (**H**,**I**) showed the location and expression of the proliferation protein PCNA (green), Ki-67 (red), and CK18 (green) in mammary gland after the browning of mammary fat (*n* = 5). Values are presented as means ± SEM, * *p* < 0.05; ** *p* < 0.01.

To determine whether the browning of mammary fat influenced the pubertal development of mice mammary glands, the fourth pair of mammary glands from mice treated with or without CL316243 were isolated for Wholemount staining and HE staining. As shown in Figure 3A, the ductal length relative to the lymph node (LN) was significantly reduced in the CL316243-treated group (Figure 3B). Meanwhile, the numbers of branch nodes (Figure 3C) and TEBs (Figure 3D) were significantly decreased with the browning of mammary fat. In addition, the percentage of the ductal area was markedly reduced by CL316243 treatment (Figure 3E). Consistently, the mammary gland weight (Figure 3F) and index (mg/g body weight) (Figure 3G) were significantly reduced after the browning of mammary gland fat. Furthermore, the immunofluorescence staining results showed a significant decline in the expression of proliferation protein PCNA in the mammary glands of mice treated with CL316243 (Figure 3H). Furthermore, the expression of proliferation marker Ki-67, co-localized with the marker of mammary epithelial cells, was greatly decreased in mammary ducts of mice treated with CL316243 (Figure 3I). Overall, these data indicated that the browning of mammary fat suppressed the development of mice mammary glands during puberty.

### 2.4. The Proliferation of HC11 Was Repressed When Co-Cultured with Brown Adipocytes

**Figure 4:**
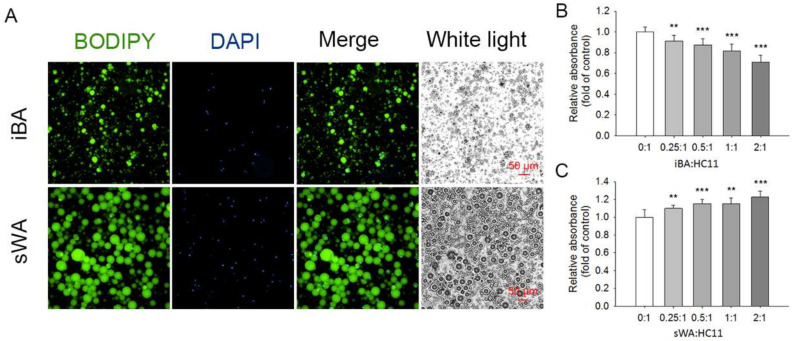
The proliferation of HC11 was repressed when co-cultured with brown adipocytes. Adipocytes from interscapular BAT (iBA) and inguinal subcutaneous WAT (sWA) were isolated from the mice during puberty and co-cultured with HC11 in different ratios (*n* = 8). (**A**) BODIPY staining showed the adipocytes (green) and the nucleus counterstained with DAPI (blue). Effects of co-culture of HC11 with iBA (**B**) or sWA (**C**) on cell proliferation of HC11. Values are presented as means ± SEM, ** *p* < 0.01; *** *p* < 0.001.

To further verify the potential regulatory effects of brown or brown-like adipocytes on mammary gland development, we performed a co-culture of the mammary epithelial cell HC11 with brown or white adipocytes to investigate the effects of brown/white adipocytes on the proliferation of HC11. The interscapular brown adipocytes (iBA) and subcutaneous white adipocytes (sWA) were isolated and purified from 8-week-old mice. BODIPY staining showed that iBA were relatively tiny and multi-locular, while sWA were relatively large and unilocular (Figure 4A). Then, HC11 cells were co-cultured with iBA or sWA in different ratios. Interestingly, we observed that the iBA with a ratio from 0.25:1 to 2:1 significantly inhibited the proliferation of HC11 (Figure 4B), while the sWA elicited the promotive effect on HC11 proliferation (Figure 4C). These in vitro observations suggested that brown adipocytes might suppress mammary gland development by repressing the proliferation of mammary epithelial cells.

### 2.5. Serum Phosphatidylcholine Level Was Elevated in Response to the Browning of Mammary Fat

**Figure 5:**
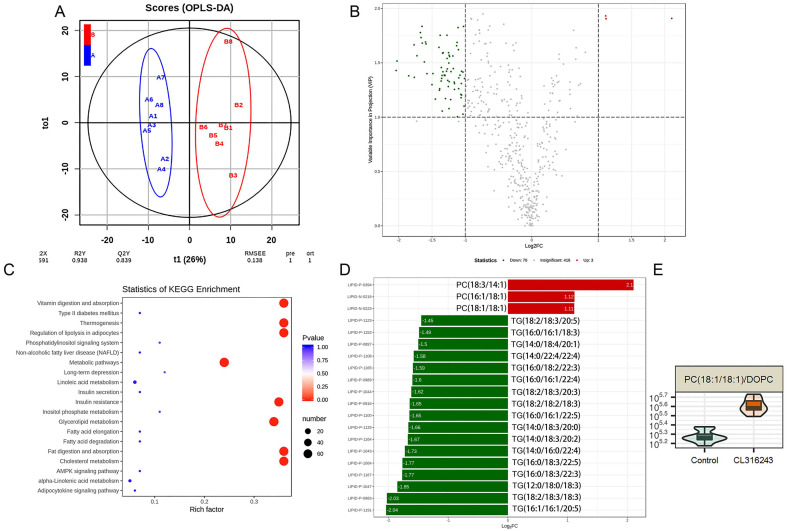
Serum phosphatidylcholine level was elevated in response to the browning of mammary fat (*n* = 8). (**A**) the Orthogonal Partial Least Squares Discriminant Analysis scores of the serum on the effect of browning of mammary fat. (**B**) a volcano plot of metabolites between the two groups. (**C**) differential metabolites involved in top 20 enrichment pathways. (**D**) the down-regulated (green bars) and up-regulated serum metabolites (red bars) of CL316243-treated mice compared with control mice. (**E**) the violin chart of DOPC, one of the differential metabolites, in control and CL316243 groups.

Given the significant suppression of mammary gland development with the browning of mammary fat by CL316243 and the repression of HC11 proliferation by co-culture with brown adipocytes, we next explored the possible underlying mechanisms by analyzing the serum metabolites with lipidomics. An Orthogonal Partial Least Squares Discriminant Analysis showed that there was a distinct separation in serum metabolites between the control and CL316243-treated groups (Figure 5A), indicating that the browning of mammary fat influenced the serum lipid profile. As shown in Figure 5B, we found 73 metabolites presented metabolic differences between the control and CL316243-treated groups, with 70 metabolites down-regulated (upper left, green dots) and 3 significantly up-regulated (upper right, red dots). Then, we mapped the metabolites to the KEGG pathways to determine whether specific pathways were enriched with metabolites in response to the browning of the mammary gland. Results showed that the metabolites were mostly enriched in metabolic pathways and thermogenesis, the regulation of lipolysis in adipocytes, insulin resistance, glycerolipid metabolism, fat digestion and absorption and cholesterol metabolism, vitamin digestion, and absorption pathways (Figure 5C). Among the differential metabolites, phosphatidylcholine (PC, 18:3/14:1), PC (16:1/18:1), and PC (18:1/18:1) (DOPC) in serum were significantly up-regulated in response to the browning of mammary fat (Figure 5D), suggesting that these up-regulated PCs might be responsible for the suppression of mammary gland development. The DOPC (Figure 5E) was selected in the following experiments.

### 2.6. DOPC Exerted an Inhibitory Effect on the Proliferation of HC11

**Figure 6:**
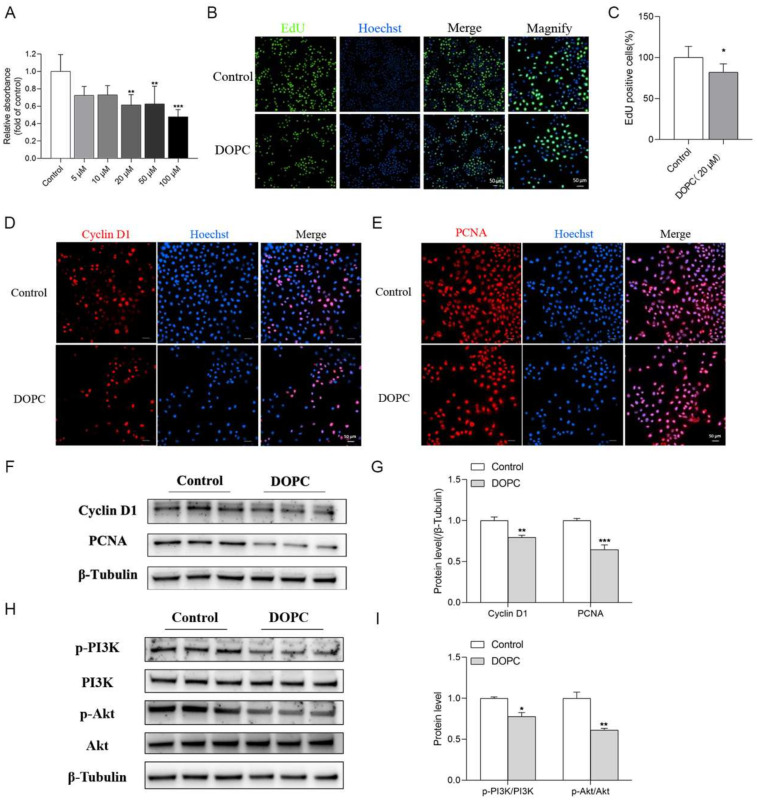
DOPC exerted an inhibitory effect on the proliferation of HC11. (**A**) CCK8 assay showed the vitality of HC11 treated with DOPC (0, 5, 10, 20, 50, 100 μM) for 24 h (*n* = 4). (**B**) EdU staining showed the effect of DOPC (20 μM) on the proliferation of HC11. (**C**) analysis of the percentage of EdU-positive cells (*n* = 5). Representative images showed the immunofluorescence staining of Cyclin D1 (**D**) and PCNA (**E**) of HC11 treated with DOPC (20 μM) for 24 h (*n* = 6). Western blot revealed the protein level of Cyclin D1 and PCNA (**F**) and PI3K and Akt (**H**) in HC11. The graph indicates the densitometry with expression ratios of Cyclin D1/β-Tubulin and PCNA/β-Tubulin (**G**), p-PI3K/PI3K, and p-Akt/Akt (**I**). Values are presented as means ± SEM, * *p* < 0.05; ** *p* < 0.01; *** *p* < 0.001.

Further, we investigated the potential effects of DOPC on the proliferation of HC11 cells and found that DOPC significantly repressed the proliferation of HC11 (Figure 6A) and the 20 μM DOPC was chosen for the subsequent studies. In agreement, the percentage of EdU-positive cells was significantly decreased after HC11 was incubated with DOPC (Figure 6B,C). In addition, immunofluorescence staining revealed that the expression of proliferative markers such as Cyclin D1 (Figure 6D) and PCNA (Figure 6E) was markedly inhibited by DOPC treatment. Similarly, the results of Western blot assays (Figure 6F) indicated that DOPC significantly suppressed the protein expression of Cyclin D1 and PCNA (Figure 6F,G). These findings indicated that DOPC exerted an inhibitory effect on the proliferation of HC11. Furthermore, we observed that DOPC led to a significant decrease in the ratios of p-PI3K/PI3K and p-Akt/Akt, indicating repression of the PI3K/Akt signaling pathway (Figure 6H,I) and suggesting the possible involvement of the PI3K/Akt pathway in this process.

### 2.7. PI3K/Akt Pathway Was Involved in the DOPC-Inhibited Proliferation of HC11

**Figure 7:**
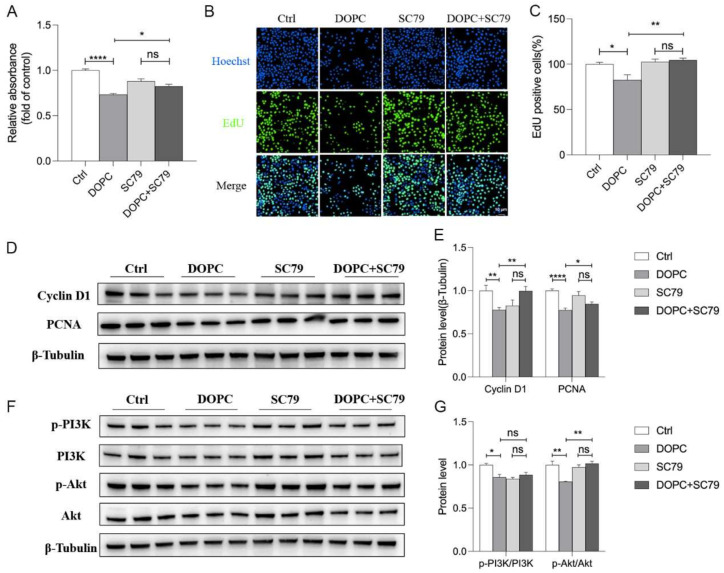
The PI3K/Akt pathway was involved in DOPC-inhibited proliferation of HC11. (**A**) the effect of SC79, an Akt activator, on the proliferation of HC11 was determined by CCK8 assay after 24 h incubation (*n* = 3). (**B**) EdU staining showed the effect of SC79 (5 μM) on the proliferation of HC11, and graph (**C**) indicated the percentage of EdU-positive cells (*n* = 3). Western blot revealed the protein level of Cyclin D1 and PCNA (**D**) and PI3K and Akt (**F**) in HC11 treated with SC79 (5 μM) for 24 h (*n* = 6). The graph indicates the densitometry with expression ratios of Cyclin D1/β-Tubulin and PCNA/β-Tubulin (**E**), p-PI3K/PI3K, and p-Akt/Akt (**G**). Values are presented as means ± SEM, * *p* < 0.05; ** *p* < 0.01; **** *p* < 0.0001; ns: not significant.

To further confirm the involvement of the PI3K/Akt signaling pathway in the DOPC-induced repression of HC11 proliferation, SC79, an Akt phosphorylation activator, was applied to activate Akt in this study. Indeed, we found that the decreased ratio of p-Akt/Akt induced by DOPC was elevated by SC79, indicating the activation of the Akt pathway (Figure 7F,G). Accordingly, we observed that the significant suppression of HC11 proliferation in response to DOPC (20 μM) was eliminated by SC79 (5 μM) (Figure 7A). Consistently, the DOPC-induced significant reduction in the percentage of EdU-positive cells was reversed by SC79 (Figure 7B,C). Furthermore, the inhibitory effects of DOPC on the expression of Cyclin D1 and PCNA were abolished by SC79 (Figure 7D,E). These results suggested that the PI3K/Akt pathway was involved in the DOPC-induced repression of HC11 proliferation. 

## 3. Discussion

In this study, we demonstrated that the development of mice mammary glands was suppressed by the browning of mammary fat during puberty, which was associated with the elevated serum DOPC and inhibition of the PI3K/Akt signaling pathway. It has been widely appreciated that the development of the pubertal mammary gland plays a critical role in the performance of lactation function [24]. In addition, numerous ducts with TEBs invade and elongate within the fat pad during pubertal, which is crucial for the structural formation of mammary glands [25]. Similarly, in the present study, significant increases in the length of ducts, number of TEBs and ductal branches, and ductal area percentage were detected in mammary glands from 3-week-old to 8-week-old mice. Traditional cognition assumed that mammary glands mainly consist of white adipose tissues. However, our results showed that brown adipose tissues exist in the mammary glands of mice and decreased significantly from 3 weeks to 8 weeks during puberty. In line with our study, it has been reported that both white adipose tissues and brown adipose tissues are present in postnatal mammary fat [26]. Similarly, studies showed that the amount of brown adipose tissue is developmentally regulated in mammary glands [27]. Furthermore, environmental factors like cold stimulation could promote the recruitment of beige adipocytes in the white adipose tissue and facilitate the transfer under certain circumstances [28].

The existence and developmental decrease in brown adipocytes in the mammary fat suggests their possible regulatory effect on pubertal mammary gland development. It has been suggested that the degree of branching complexity and epithelial differentiation was negatively regulated by the brown adipocytes [29]. Indeed, we found that the induction of mammary fat browning by CL316243 treatment significantly suppressed the pubertal development of mice mammary glands, with decreased ductal length and area, numbers of branch nodes, and TEBs. Consistent with the observations in morphology, the weight and index of mammary glands were significantly reduced; additionally, the expression of the proliferation protein PCNA was decreased with the treatment of CL316243. These results highlighted the inhibitory effects of mammary fat browning on pubertal mammary gland development. Similarly, it has been demonstrated that there is an epithelial differentiation in mammary glands induced by the depletion of mammary BAT in early postnatal development, implying the regulation of brown adipocytes on the differentiation of mammary epithelial cells during the outgrowth of ducts in prepuberty [14]. On the other side, Gouon-Evans et al. reported that the number of TEBs and branches, as well as the ductal lengths, in mice mammary glands were not affected by the injection of CL316243 from 12 d to 28 d of age [14]. These discrepancies between our and Gouon-Evans’s results might be due to the different time points of the CL316243 injection. In our study, the pharmacological induction by intraperitoneal injection of CL316243 to mice was from 4 weeks to 8 weeks, during which the mammary gland entered a rapid development stage [27]. 

Based on the findings that the browning of mammary fat repressed the pubertal mammary gland development, we further examined the direct effects of brown adipocytes on the proliferation of mammary epithelial cells. Interestingly, the co-culture of the interscapular brown adipocytes and HC11 with a ratio from 0.25:1 to 2:1 significantly inhibited the proliferation of HC11. These data clarified that the brown adipocytes repressed the proliferation of HC11.

Given that the proliferation of HC11 was repressed by brown adipocytes and the development of the pubertal mammary gland was suppressed by the browning of mammary fat, we further explored the possible underlying mechanisms using LC-MS/MS, especially the potential contribution of lipid metabolites. By analyzing the serum lipid profiles, we found that only three kinds of phosphatidylcholine (PC) were significantly increased among the 73 significantly different metabolites, suggesting that the up-regulated PCs might influence the development of mammary glands. PC, the major phospholipid component of eukaryotic membranes, as well as choline metabolites derived from its synthesis and catabolism, are critically involved in multiple biological processes such as cell proliferation, survival, and metabolism [30]. It has been demonstrated that the proportion of sn-2-arachidonoyl-phosphatidylcholine (20:4-PC) increases significantly during the G1 phase of the cell cycle [31,32]. Moreover, PC also contributes to the transformation of brown-like adipocytes [33], which supported our results that the PC was up-regulated with the browning of mammary fat along with an increase in the BAT index in the CL316243 group. PC, composed of glycerol backbone, long-chain fatty acids, phosphate groups, and choline molecules, is also involved in the proliferative growth of mammary epithelial cells [34]. The long-chain fatty acids could be transported and absorbed by the mammary gland via circulation, thus subsequently consuming the phosphatidylcholine [35,36]. In our results, dioleoylphosphocholine (DOPC), one of the differential phosphatidylcholines in serum, was significantly up-regulated in response to the browning of mammary fat. As we speculate, the sharp increase in DOPC induced by the browning of mammary fat might exceed the capacity of epithelial cells to absorb and utilize. As a result, the DOPC was accumulated in circulation, which might inhibit the proliferation of mammary epithelial cells and thus showed a suppression effect on mammary glands.

We further investigated whether DOPC modulated the suppression of mammary gland development by inhibiting mammary epithelial cell proliferation. Interestingly, our findings revealed that DOPC significantly repressed the proliferation of HC11, along with a decreased expression of proliferative markers (CyclinD1 and PCNA) [37,38]. In line with our results, *sn*-2-arachidonoyl-phosphatidylcholine, one of the phosphatidylcholines, has been reported to delay cell cycle progression [31]. The inconsistent effects of phosphatidylcholine on cell proliferation probably result from the difference in their biological structure and the distinct cell types [39].

It has been well-documented that the PI3K/Akt pathway plays a vital part in cell survival, proliferation, and metabolism [40,41]. In the present study, we found that DOPC inhibited the activation of the PI3K/Akt pathway, while the activator of Akt reversed the inhibitory effects of DOPC on HC11 proliferation. Our results were consistent with the reports that phosphatidylcholine also plays an important role in cell signaling transduction as the phosphatidylcholine transferase could act as a carrier to transport phosphatidylcholine from membrane to membrane [42]. Additionally, increasing the cellular ratio of phosphatidylcholine (20:4-PC) diminished Akt phosphorylation and suppressed cyclin expression [31]. These findings suggested that the PI3K/Akt pathway was involved in the DOPC-induced repression of HC11 proliferation. Consistently, it has been shown that the PI3K/Akt pathway participates in the stearic acid-suppressed [4] and lauric acid-stimulated [7] proliferation of HC11 cells. Similarly, the PI3K/Akt signaling pathway is essential for the survival of the mammary epithelial cell population in the rapidly expanding alveolar during pregnancy [41].

**Figure 8:**
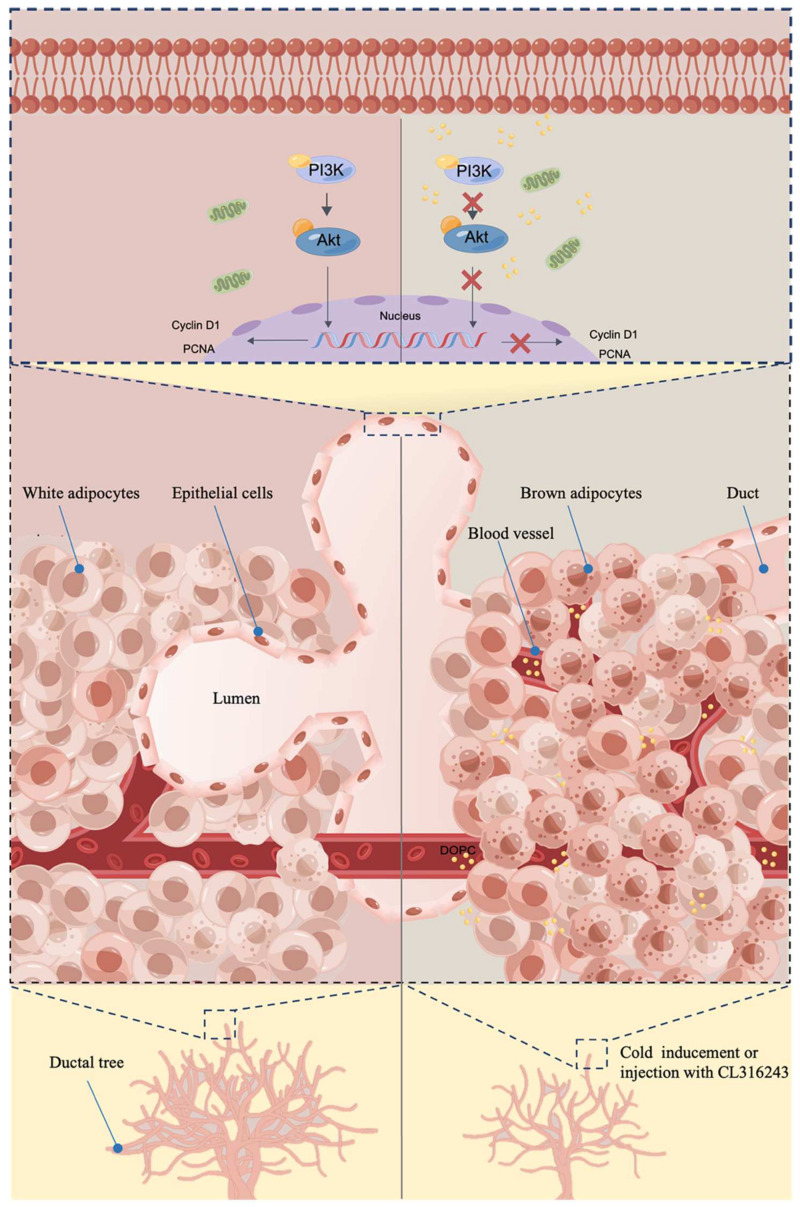
A potential mechanism underlying the suppression of pubertal mammary gland development induced by the browning of mammary gland. (By Figdraw, www.figdraw.com, accessed on 14 November 2022).

To sum up, we found that brown adipocytes in mammary fat decreased with the development of mice. In the meanwhile, the browning of mammary fat by injection of CL316243 suppressed the pubertal development of mammary glands, accompanied by a significant elevation of serum DOPC. In addition, the proliferation of HC11 was repressed when co-cultured with the brown adipocytes isolated from pubertal mice or treated with the altered serum lipid metabolite DOPC. Furthermore, DOPC suppressed the activation of the PI3K/Akt pathway, while the DOPC-inhibited HC11 proliferation was reversed by SC79, an Akt activator, suggesting the involvement of the PI3K/Akt pathway in the DOPC-inhibited proliferation of HC11. Taken together, the browning of mammary fat suppressed the development of the pubertal mammary gland, which was associated with the elevated serum DOPC and the inhibition of the PI3K/Akt pathway. (Figure 8). These results provided a potential new target for the modulation of mammary gland development. In addition, these findings probably put forward a possibility that the induction of mammary fat browning might have potentially beneficial effects on treating breast cancer in the future.

## 4. Materials and Methods 

### 4.1. Animals

50 C57BL/6J female mice (3-week-old) purchased from the Animal Center of Medical Laboratory in Guangdong were randomly divided into three groups after a three day pre-feed period, and then were sacrificed at 3 weeks (*n* = 10), 5 weeks (*n* = 10), and 8 weeks (*n* = 10) separately. For the browning of mammary fat studies, mice were treated with either CL316243 (β3-adrenoceptor agonist) (*n* = 10) dissolved in physiological saline or vehicle (physiological saline) (*n* = 10) via intraperitoneal injection once daily with 1 mg/kg from 4 weeks to 8 weeks. All mice were maintained at 25 ± 1 °C with 60 ± 5% relative humidity under a 12 h light/dark cycle with free access to water and food. The body weight and food intake were recorded every week during this period. In the meanwhile, the rectum temperature of mice was detected by a digital thermometer (BAT-12, Physitemp Instruments, Clifton, NJ, USA). The study was approved by the College of Animal Science, South China Agricultural University (Permit Number SYXK(Yue) 2022-0136, Permit Date 13 January 2022). All experiments were conducted in accordance with The Instructive Notions with Respect to Caring for Laboratory Animals (Ministry of Science and Technology, Beijing, China).

### 4.2. Wholemount and Hematoxylin and Eosin (H&E) Staining

One side of the fourth pair of mammary glands in mice was taken and fixed on a slide in Carnoy Fixative overnight, immersed in acetone for 12–24 h and rinsed with gradient ethanol, and then stained with carmine alum for 4–12 h. The slides were immersed in a solution with 2% hydrochloric acid −70% ethanol for 24 h and rinsed with gradient ethanol and then xylene dehydration overnight, sealed, while the paraffin-embedded mammary gland sections (5 μm) were stained with hematoxylin and eosin for H&E staining. Images were photographed using a Nikon Eclipse Ti-s microscope (Nikon Instruments, Tokyo, Japan).

### 4.3. Immunohistochemistry and Immunofluorescence Staining

The slides were immersed with dimethylbenzene for 15 min (three times) and rinsed in ethanol with different concentrations (5 min each), and then antigen retrieval was performed in the solution of sodium citrate. After that, slides were permeabilized with 0.3% Triton X-100 for 10 min, blocked with 3% BSA for 30 min, and incubated with a primary antibody at 4 °C overnight. Then, they were incubated with the secondary antibody for 1 h. For the immunohistochemistry detection, the nuclei were stained with 4′,6-diamidino-2-phenylindole. For immunofluorescence assay, slides were dehydrated with an ethanol gradient, cleared in xylene, and then sealed finally. All the images were captured using upright microscopes. 

### 4.4. Real-Time Quantitative PCR

The total RNA of mammary glands was extracted using the Hipure Universal RNA Mini kit (R4130-02, Magen, Shanghai, China) according to the manufacturer’s protocol. In total, 1μg RNA was mixed with 2 μL gDNA remover for 5 min at room temperature and then we added the 4x RT Master Mix and Nuclease-free ddH_2_O according to the manufacturer’s instructions. qRT-PCR was performed with the SYBR Green Real-time PCR Master Mix reagents using the QuantStudio 3 Flex Real-Time PCR system. *β-actin* was used for normalization, and the relative expression of genes was calculated using the 2^′△△CT^ method. Primer sequences are listed in Table 1.

### 4.5. Cell Culture and Treatments

The interscapular BAT and subcutaneous WAT were excised and minced from mice during puberty and digested with the type I collagenase at 37 °C for 30 min. Then, cells were filtered with a 70 µm cell strainer before being centrifuged at 500× *g* for 5 min. The interscapular brown adipocytes (iBA) and subcutaneous white adipocytes (sWA) were identified by BODIPY staining and co-culture with HC11 in different ratios. Cells were cultured in RPMI-1640 medium with 10% FBS, 100 U/mL penicillin, and 100 μg/mL streptomycin. When analyzing the effects of iBA and sWA on the proliferation of HC11, the iBA and sWA cells were removed from the medium of HC11 after being co-cultured for 24 h, and then 100 μL fresh medium with 10 μL CCK8 solution was added to HC11 cells and incubated for 1 h before detection.

### 4.6. CCK8 Assay and EdU Incorporation Assay

HC11 cells were seeded into a 96-well plate with a density of 8000 cells per well and treated with DOPC (20 μM) with or without SC79 (5 μM) (the activator of the Akt signaling pathway) for 24 h, and then incubated with 100 μL RPMI-1640 medium containing 10 μL CCK8 solution at 37 °C for 1 h before detection by a Synergy 2 Multi-Mode Reader (Bio Tek Instruments, Inc., Winooski, VT, USA). While for the EdU incorporation assay, cells were incubated with EdU reagent at 37 °C for 2 h and were then fixed in 4% paraformaldehyde for 15 min and washed with PBS 3 times (5 min each). After that, cells were permeabilized with 0.3% Triton X-100 for 10 min and washed with PBS before adding a click additive solution for 30 min. The nuclei were stained with Hoechst, and images were captured with a Nikon Eclipse Ti-s microscope (Nikon Instruments, Tokyo, Japan).

### 4.7. Western Blot

Tissue and cells were lysed with RIPA buffer (P0013B, Beytime, HangZhou, China) containing 1 mM PMSF (ST505, Beyotime, China). The concentration was detected using a BCA kit (Thermo Fisher Scientific, Waltham, MA, USA), and total protein was separated by SDS-PAGE and then transferred to PVDF membranes (0.45 µm; Millipore, Darmstadt, Germany). After blocking with TBST containing 5% nonfat milk at room temperature for 2 h, membranes were incubated overnight at 4 °C with the following primary antibodies: UCP1, PI3K, p-PI3K, Akt, p-Akt, Cyclin D1, PCNA, followed by an HRP-conjugated secondary antibody (Bioworld) at room temperature. Proteins on the membranes were detected using a FluorChem M Fluorescent Imaging System (Protein Simple, San Jose, CA, USA) and analyzed by Image J software (version: 1.51, National Institutes of Health, Bethesda, MD, USA).

### 4.8. Body Composition and Infrared Thermography

After treatment with CL316243 for 4 weeks, the lean and fat content of mice was detected by Small Animal Body Composition Analysis and Imaging System NMR Analyzer (MesoQMR23-060H, Niumag Corp., Shanghai, China). Then, the iBAT temperature of mice under an environmental temperature of 25 °C or a cold exposure temperature of 4 °C for 4 h was determined by FLIR Quick Report software (version: 4.40.1, FLIR ResearchIR Max; FLIR System).

### 4.9. Lipidomics Analysis

The serum sample of mice after being treated with CL316243 for 4 weeks was analyzed by ultra performance liquid chromatography (UPLC) and tandem mass spectrometry (MS/MS) (Wuhan Metware Biotechnology, Wuhan, China).

### 4.10. Statistical Analysis

All values are expressed as the mean ± standard error of mean (SEM). Differences between two groups were analyzed with unpaired Student’s *t*-tests, while differences among three or more groups were compared with one-way ANOVA, and *p* < 0.05 was considered significant.

## Figures and Tables

**Table 1:** The primer sequences for the amplification of *UCP1* and *β-actin*.

Gene	Primer	Primer Sequence (5′–3′)
*UCP1*	Forward	ATTCAGAGGCAAATCAGCTTTG
	Reverse	GTGTTTCTCTCCCTGAAGAGAA
*β-actin*	Forward	GGTCATCACTATTGGCAACGAG
	Reverse	GAGGTCTTTACGGATGTCAACG

## Data Availability

Data are contained within the article.

## Abbreviations

DOPC: dioleoylphosphocholine; TEBs, terminal end buds; UCP1, uncoupling protein 1; iBA, interscapular brown adipocytes; sWA, subcutaneous white adipocytes; UPLC, ultra performance liquid chromatography; SEM, standard error of mean; BAT, brown adipose tissue; LN, lymph node; PC, phosphatidylcholine.
